# Synthesis and Characterisation of a Monolithic Imprinted Column Using a Methacrylic Acid Monomer with Porogen Propanol for Atenolol Analysis

**DOI:** 10.1155/2020/3027618

**Published:** 2020-02-28

**Authors:** Aliya Nur Hasanah, Firdha Senja Maelaningsih, Fadli Apriliandi, Akhmad Sabarudin

**Affiliations:** ^1^Pharmaceutical Analysis and Medicinal Chemistry Department, Faculty of Pharmacy, Universitas Padjadjaran, Jalan Raya Bandung Sumedang KM 21, 5, Sumedang, Indonesia; ^2^Chemistry Department, Universitas Brawijaya, Malang, Indonesia

## Abstract

A monolithic imprinted atenolol column was constructed by in situ polymerisation using a methacrylic acid monomer and a 1 : 1 (v/v) porogen of propanol: toluene with two template: monomer: crosslinker combinations, namely, MIP 1 (1 : 4 : 20) and MIP 2 (1 : 5 : 20). Physical characterisation of the monolithic columns consisted of permeability testing, Fourier transform infrared (FTIR) testing, surface area analysis (SAA), and scanning electron microscopy (SEM). The permeability value of four monolithic columns was in the good category: MIP 1 (24.01 mD), NIP 1 (56.43 mD), MIP 2 (23.03 mD), and NIP 2 (14.47 mD). The polymerisation process of these four monolithic imprinted columns was carried out perfectly, as shown by the absence of vinyl groups (1000 cm^−1^ and 900 cm^−1^) during FTIR testing. Based on SAA testing, the pores of the four polymers were classified as mesopores. The best monolithic column was MIP 1, as seen from the intercolumn and intracolumn reproducibility values and a % RSD <2.0%. The MIP 1 column was selective towards atenolol, as seen from the selectivity factor, imprinting factor (IF), and resolution (Rs) values. The IF values of MIP 1 were atenolol (204.62), metoprolol (3.36), and propranolol (1.27). The Rs value between atenolol and the analogue compounds was 7.23. The MIP 1 column can be used for the analysis of atenolol in blood serum samples with an average percentage recovery rate of 94.88 ± 4.43%.

## 1. Introduction

Beta blockers have become the first choice for hypertension treatment over the past four decades [1] to reduce blood pressure, heart rates, anxiety, and muscle tremors. Currently, beta blockers are often misused in certain sports that do not require excessive physical activity, such as archery, billiards, golf, skiing, and underwater sports. The effect caused by taking these drugs is the slowing of the average heart rate such that one becomes calmer and more focussed [2]. The International Olympic Committee includes beta blockers in the doping category because they improve performance in athletes [3]. Atenolol is a beta blocker with the chemical name 4-(2-hydroxy-3-[(1-methyl ethyl) amino] propoxy) benzene acetamide, which is effective in cardiovascular therapy such as hypertension, angina, and arrhythmias [4]. The long-term use of atenolol can increase morbidity and mortality in patients with hypertension as compared with other antihypertensive agents [5]; therefore, monitoring the use of atenolol is necessary, especially for abuse as doping.

Evidence of doping in athletes can only be obtained through examination of drug metabolites found in urine, blood, and other biological samples. Atenolol levels in the blood are very low, at 600 ng/mL three hours after the first dose and 50–70 ng/mL after 24 hours [6]. One SPE technique that is selective and able to eliminate the interfering matrix and concentrate analytes for detection is molecularly imprinted polymer (MIP) technology [7–9].

Current atenolol analysis techniques are spectrophotometry [10], electrophoresis [11], and chromatography [12]. In addition, the availability of various columns makes HPLC the most commonly used and acceptable technique for researchers [13]. Conventional HPLC still uses particle columns as stationary phases, which require large amounts of mobile phase. Moreover, another disadvantage of this type of column is poor mass transfer and the requirement for high pressure. The column particle pressure must be approximately 450−500 bar [14].

Monolithic columns can be constructed using molecularly imprinted polymer (MIP) technology to increase selectivity. The MIP is designed to have selectivity for the target at the molecular level by possessing special binding sites that are more specific to certain analytes [15, 16]. In addition, the polymers produced by MIPs are more stable, stronger, and resistant to pH, solvent, and temperature [17].

The components used for the construction of imprinted monolithic columns are functional monomers, crosslinkers, porogens, and initiators. The functional monomer used in the present study was methacrylic acid (MAA), which is a universal monomer most often used for the manufacture of monolithic columns [18]. In addition, atenolol is alkaline, based on a pKa value of 9.6 [19]; thus, the imprinted atenolol column can be synthesised using MAA. MAA undergoes a dimerisation reaction that can increase the imprinting effect; the high molar fraction of MAA produces a large porous polymeric material that increases polymer-binding capacity [20]. Propanol, as a porogen in the manufacture of monolithic columns, has a low dielectric constant; therefore, it tends to be nonpolar. Nonpolar or less polar porogens increase the formation of functional template-monomer complexes because hydrogen bonding is facilitated, which increases imprinting efficiency [16, 21].

To the best of our knowledge, a monolithic column for atenolol analysis has not yet been manufactured. A research study about the monolithic column for beta blockers based on imprinted technology was only published by Zhang et al. [22] for propranolol. In the present study, an imprinted monolithic column was constructed using methacrylate acid (MAA) as a functional monomer and propanol as a porogen. Moreover, permeability testing was performed, followed by physical characterisation using scanning electron microscopy (SEM), Fourier transform infrared (FTIR) testing, and surface area analysis (SAA) using the Brunauer, Emmett, and Teller (BET) method. Furthermore, analytical performance for atenolol selectivity with respect to analogue compounds, reproducibility testing, and application of atenolol analysis in serum using HPLC was carried out.

## 2. Materials and Methods

### 2.1. Materials

3-(Trimethoxysilyl)-propyl methacrylate (MAPS) (Tokyo Chemical Industry), atenolol (Tokyo Chemical Industry), metoprolol tartrate hydrochloride (Tokyo Chemical Industry), propranolol hydrochloride (Tokyo Chemical Industry), acetic acid, phosphoric acid, hydrochloric acid (HCl), acetone (Merck), benzoyl peroxide (BPO), ethanol proanalysis, sodium hydroxide, pyridine, and propanol were from Merck. Methacrylate acid (MAA), triethylamine, and ethylene glycol dimethacrylate (EGDMA) were provided by Sigma-Aldrich. Aquabidestillata (Ipha Laboratories), methanol (JT Baker and Fisher Scientific), toluene (Mallinckrodt), and acetonitrile were of HPLC gradient grade (Fisher Scientific). All materials other than those mentioned were of proanalysis grade.

A BET surface area analyser (NOVA instrument© 1994–2010), field emission-scanning electron microscope (FE-SEM) (FEI Quanta 650 FEG), Fourier transform infrared (FTIR) apparatus (IR-Prestige-21 Shimadzu), silicosteel column (outer diameter 1/16 mm, inner diameter 1.02 mm, length 10 cm) (Tubing Sulfinert Restek), oven (Memmert), centrifuge (Labnet C2500-R Prism R™), scanning electron microscope (SEM) (Hitachi TM3000), ultra high-performance liquid chromatography unit (Waters Acquity™), ultrasonicator (NEY 19H), water bath (Memmert), and glass tools were commonly used in laboratories.

### 2.2. Silanisation Process

Silicosteel column preparation was carried out by silanisation referring to the procedure described by Shu et al. [23]. First, the silicosteel column was washed with a syringe containing aquabidestillata, filled with 0.2 M NaOH solution for 30 minutes, and repeated. Subsequently, the column was washed three times with aquabidestillata, filled with 0.2 M HCl for 30 minutes, repeated, washed again three times with aquabidestillata, and finally washed with acetone. The silicosteel column was then filled with a mixture of 30% MAPS in acetone and pyridine at a ratio of 30 : 65 : 5 of MAPS : acetone : pyridine. Both ends of the column were closed, and it was left at room temperature for 12 hours. The process of silanisation with MAPS was repeated twice. Following silanisation, the silicosteel column was washed with acetone and cut into 10-cm pieces [23].

### 2.3. Synthesis of Imprinted Monolithic Columns

#### 2.3.1. Prediction of Template-Monomer Interaction

To predict the interaction between the template and monomers (MAA), a computational approach was used. Two-dimensional structures of MMA and ITA were drawn and then converted into a three-dimensional structure using ChemBio3D Ultra 12.0 program. Geometry optimisation was then carried out with the *ab initio* method (Hartree Fock, base set 3-21G) using the games interface on the ChemBio3D Ultra 12.0. The docking process between MAA and ITA was undertaken using PyRx software–virtual screening tools with AutoDock Vina. The docking results were analysed by comparing the position and type of bond formed and the value of the binding affinity of the template and MAA using AutoDockTools 1.5 software.

#### 2.3.2. Synthesis of Imprinted Monolithic Columns

Monolithic column synthesis used an in situ polymerisation technique with a mixture of atenolol as a template, MAA as a functional monomer, and EGDMA as a crosslinker, with two variations of template:monomer:cross-linker (1 : 4 : 20 and 1 : 5 : 20). The mixture of the template, monomer, and porogen propanol (1.5 mL) was homogenised for 5 minutes using an ultrasonicator, followed by the addition of a BPO radical initiator (1.55 mmol; 0.125 g) and homogenisation for a further 30 minutes. The homogenate was inserted into the liquidated silicosteel column using a syringe; both ends of the column were closed, and it was polymerised in an oven at 80°C for 18 hours. The composition of the material used for the synthesis of the imprinted monolithic columns can be seen in Table 1.

#### 2.3.3. Template Removing from Imprinted Monolithic Columns

After the synthesis process, the monolithic column was washed with the methanol : acetic acid (90 : 10 v/v) mobile phase at a flow rate of 0.01–0.03 mL/minute to remove the residual unreacted substance and atenolol template. The mobile phase that passed through the monolithic column was accommodated and then monitored using an ultraviolet (UV) spectrophotometer by identifying absorption peaks at the maximum wavelength of the UV spectrum as compared with the atenolol standard.

### 2.4. Physical Characterisation

The imprinted monolithic column permeability test was carried out, followed by physical characterisation using a SEM to view microscopic structures, FTIR to determine the functional groups, and SAA using the BET method to determine pore size.

#### 2.4.1. Permeability Testing

The permeability test for the imprinted monolithic column was carried out by measuring the pressure drop of the ethanol mobile phase at a constant flow rate of 0.05 mL/minute, followed by calculation using the Darcy equation [24]:

(1) $$\begin{array}{c} K=\frac{\eta Lu}{\Delta p}=\frac{\eta LF_{m}}{\Delta p\pi r^{2}}, \end{array}$$

where *K* is the permeability (m^2^), *η* is the mobile phase viscosity (Pa·s), *L* is the column length (m), ∆*p* is the pressure drop (Pa), *u* is the linear rate of the mobile phase (m/s), Fm is the flow rate of the mobile phase (m^3^/s), and *r* is the radius of the column (m).

#### 2.4.2. SEM

Physical characterisation to determine the morphology was carried out using scanning electron microscopy with energy-dispersive spectroscopy (SEM-EDS) on monolithic polymers from the summarised columns (column silanisation method followed the procedure in Section 3.2.1). One end of the monolithic column (±1 cm) was cut, and surface monolithic morphology was observed using SEM at a magnification of 2,500x [25].

#### 2.4.3. FTIR

Imprinted monolithic column functional group determination was carried out using FTIR spectroscopy by observing the infrared spectrum at wave numbers 4000−400 cm^−1^. A mass of 200 mg potassium bromide (KBr) was used as a blank, and 2 mg samples of monolithic polymer were mixed with 198 mg KBr, each of which was printed into plates and analysed using FTIR.

#### 2.4.4. SAA

Determination of the pore size and pore distribution of the unionised imprinted monolithic column material was carried out by the BET method using the SAA instrument. The principle of this method is adsorption of nitrogen gas on the surface of the monolithic column using the melting point of the monolith and certain pressures to form a monolayer followed by a multilayer. This method was carried out by dry destruction at 130°C outgas temperature [25].

### 2.5. Characterisation of Analytical Performance

The analytical performance of the imprinted monolithic columns was evaluated by counting the retention factor and number of theoretical plates with the optimal flow rate and composition of the mobile phase. Variation in the flow rate was 0.01–0.05 mL/minute. The mobile phase composition was a mixture of methanol and triethylamine (TEA), with methanol concentrations of 10−90% v/v and pH values ranging from 3 to 9.

### 2.6. Selectivity of Imprinted Monolithic Columns

Determination of the selectivity of the imprinted monolithic columns was carried out using atenolol analogues that have similar structures and properties, such as propranolol and metoprolol. Atenolol, propranolol, and metoprolol at a concentration of 10 ppm were analysed by HPLC using the imprinted monolithic column stationary phase at the most optimal HPLC conditions (flow rate and mobile phase composition). Furthermore, the selectivity factor (*α*), imprinting factor, and resolution values were calculated using the following formulas:

Selectivity factor formula:

(2) $$\begin{array}{c} \alpha =\frac{k_{1}}{k_{2}}, \end{array}$$

where *α* is the selectivity factor, *k*_1_ is a retention factor of atenolol, and *k*_2_ is a retention factor of atenolol analogues (metoprolol, propranolol).

Imprinting factor formula:

(3) $$\begin{array}{c} \text{IF}=\frac{k_{\text{MIP}}}{k_{\text{NIP}}}, \end{array}$$

where IF is the imprinting factor, *k*_MIP_ is a capacity factor of MIP, and *k*_NIP_ is a capacity factor of NIP.

### 2.7. Reproducibility Testing

Intra- and intercolumn reproducibility testing were carried out by calculating the percentage value of relative standard deviations (% RSD) of retention time, area (AUC), and retention factor of atenolol. The RSD value of each of these parameters must be less than 2.0% [26].

### 2.8. Application of Imprinted Monolithic Columns

A volume of 200 *µ*L standard atenolol solution was added to 300 *µ*L serum, followed by the addition of 1.5 mL acetonitrile and centrifugation for 15 minutes at 2,000 rpm and 10°C [27]. The supernatant was taken and filtered using a 0.45-*µ*m membrane filter, and the analyte content was measured by HPLC using the imprinted monolithic column as the stationary phase.

## 3. Results and Discussion

### 3.1. Silanisation Process

**Figure 1:**
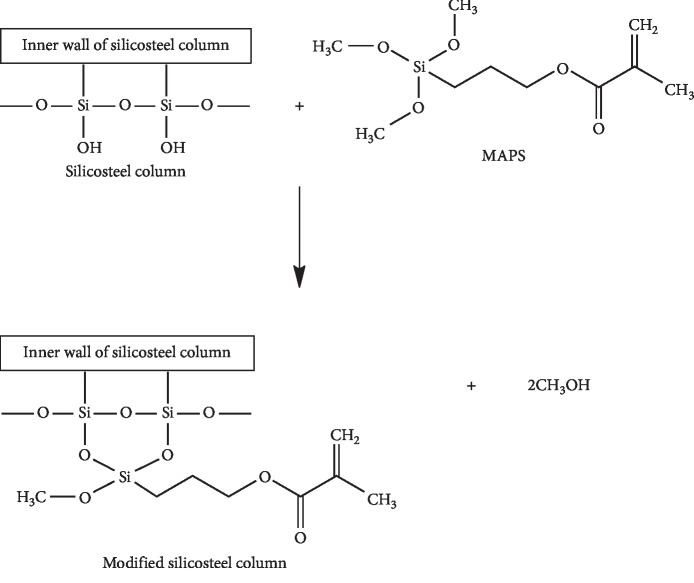
Silanisation reaction of the silicosteel column with MAPS [28].

The silanisation process in the silicosteel column was carried out prior to the synthesis of the monolith by hydrolysing the column using acidic and basic solutions (HCl and NaOH) and adding the MAPS to facilitate the formation of covalent bonds between the monolithic polymers and the silicosteel columns. The silanisation reaction that occurs between a silanol group and a methoxy group of MAPS compounds on the wall surface of the silicosteel column is shown in Figure 1. The reaction between MAPS and the silicosteel column plays an important role in the formation of in situ polymers, such that the bond between the monomer and the crosslinker becomes strong and keeps the polymer in the silicosteel column [28, 29].

### 3.2. Synthesis of Imprinted Monolithic Columns

#### 3.2.1. Prediction of Template-Monomer Interaction

**Figure 2:**
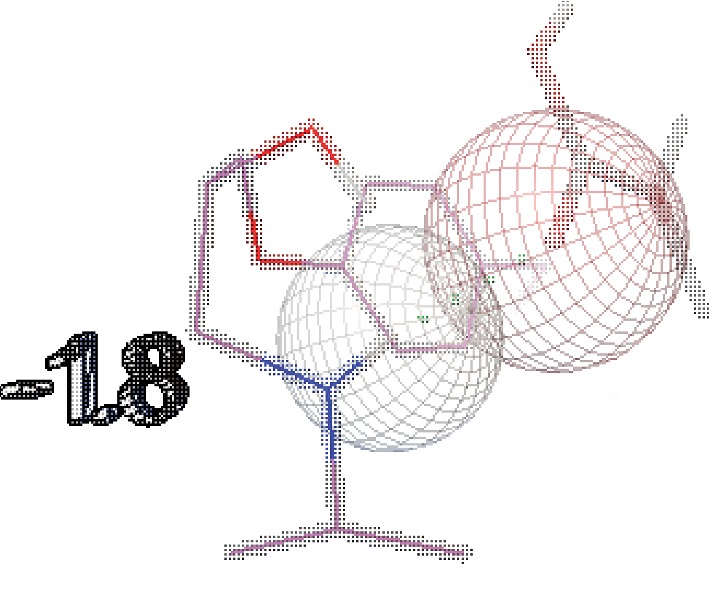
Interaction between atenolol and MAA.

Construction of imprinted monolithic columns was carried out through in situ polymerisation by dissolving atenolol as a template in porogen. Selection of the correct functional monomer is one of the most important factors in facilitating the interaction between the template and substrate. Functional monomers play an important role in increasing binding sites to maximise the formation of complexes between templates and monomers. Interactions that occur between MAA and atenolol monomers are noncovalent, rendering removal of templates easier. The interaction between atenolol and MAA can be seen using Autodock Tools software, as shown in Figure 2.

**Figure 3:**
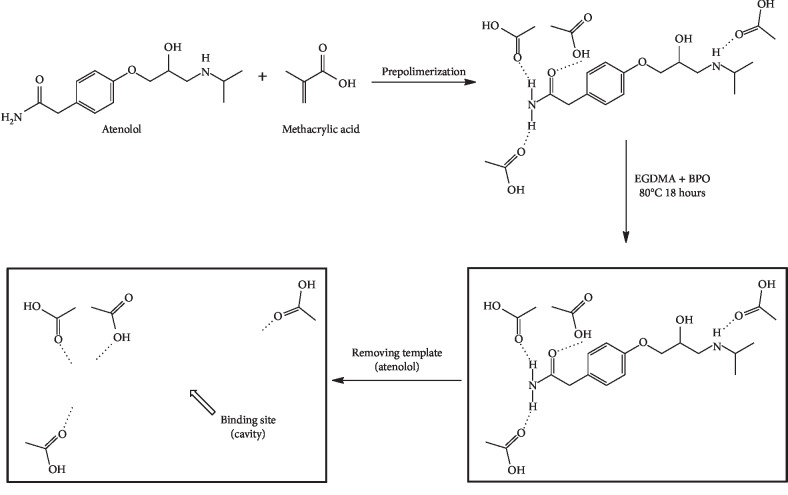
Illustration of the synthesis of the imprinted monolithic columns.

Based on previous research, bonding between atenolol and MAA is a hydrophobic interaction [22]. However, according to our prediction using a computational approach (Figure 2), the interaction between atenolol and MAA monomers is a hydrogen bond with a binding affinity of −1.8 kcal/mol. These interactions need to be further studied using other approaches. Hydrophobic interactions and hydrogen bonds produce specific binding sites for atenolol. An illustration of the polymerisation reaction that occurs is shown in Figure 3. Higher compositions of a monomer than a template will increase the polymer adsorption ability of target molecules because the number of noncovalent interactions will increase [30]; therefore, two variations of template:monomer:crosslinker (1 : 4 : 20 and 1 : 5 : 20) were constructed.

#### 3.2.2. Synthesis of the Imprinted Monolithic Columns

Selectivity is strongly influenced by the type and number of crosslinkers used in the synthesis of imprinted polymers. A high crosslinker ratio will typically result in small polymer pores and produce permanent materials with adequate mechanical stability [31]. Polymerisation occurs by chain growth reactions between two different monomers to form a series of random copolymers. The initiator greatly influences the monolith porosity reached during the copolymerisation process. In addition, monoliths will not undergo a polymerisation reaction again if the temperature is incapable of initiating a reaction in which the initiator will experience thermal decomposition to form a radical. BPO initiators will decompose to form radicals at a temperature of 80−95°C. Free radicals and unpaired electrons will react with monomers to form long polymer chains [32]. The polymerisation reaction generally lasts 16−48 hours, and the polymerisation time affects the morphology of the produced polymer [33]; therefore, the chosen polymerisation conditions were 80°C for 18 hours.

The polymerisation process consists of three stages: chain initiation (free radical formation from the initiator), carbon chain extension, and chain termination (radicals form stable molecules) [34]. Increasing the polymerisation time makes the polymer structure more rigid and facilitates the formation of molded cavities with a better shape [33]. The polymer produced following a polymerisation time of 8 hours is slightly easier to destroy as than that produced following polymerisation for 18 hours. The reduction in polymerisation time to 8 hours affects, but not significantly, the quality of the produced polymer. This is because of the fact that at the termination stage, the BPO initiator works after 8 to 24 hours; if the reaction is no longer than 24 hours, the produced polymer is relatively stable [35]. Therefore, the polymerisation time used here was 18 hours.

Many factors influence the quality of monolithic polymers, one of which is the porogen. Porogens are very important in the formation of pores within polymers. The nature and level of porogen solubility will determine the strength of noncovalent interactions and affect polymer morphology and MIP performance. Porogen solvents must be able to dissolve all MIP components (templates, monomers, cross linkers, and initiators). Porogens must produce large enough pores such that the flow properties of the polymer are good and must also have relatively low polarity to reduce interference during the formation of template and monomer complexes, increasing MIP selectivity. Porogens with low-solubility phases separate earlier and tend to have larger pores and a lower surface area, whereas porogens with higher solubility phases will separate later while polymerising and produce smaller pore sizes and a larger surface area [31, 36].

In a previous study synthesising MIP with MAA monomers using methanol as a porogen, the resulting adsorption capacity values were still low because the bonds between the analyte and template were not strong [9]. The choice of propanol as a porogen is based on the lower polarity (dielectric constant = 20.1) than methanol (dielectric constant = 33.0); thus, macropores are not formed, and hydrogen bonding is facilitated. Based on optimisation results of the composition of the monolithic column, propanol produces polymers that are easily destroyed; therefore, the pressure from the monolithic column becomes large. Porogens are related to pore formation. A single porogen produces a single pore, micropore, mesopore, or macropore, whereas a porogen mixture produces a combined pore, namely, micropore-mesopore, mesopore-macropore, or micropore-macropore [36]. The type of the pore expected in monoliths is a mixture of macropores and mesopores. Macropores play a role in convective mass transfer, whereas mesopores act as a provider of the adequate surface area for interactions between analyte and stationary phases, increasing the binding capacity to target molecules [28].

Destructive polymers are caused by the formation of only one type of pore size because the polymer density is increased. An increased propanol content will increase column efficiency but produce higher back pressure [23]; therefore, a porogen mixture of propanol and toluene was used. Toluene was chosen because it is nonpolar (dielectric constant = 2.38) and will stabilise hydrogen bonds and increase printing efficiency [16, 31]. Based on the optimisation results, the best porogen composition was a mixture of propanol and toluene (1 : 1). The best polymers have a shape that is neither easily broken nor too hard and porous.

#### 3.2.3. Template Removing from Imprinted Monolithic Columns

Following the synthesis of the imprinted monolithic column, it was washed to remove residual substances and atenolol templates. A solvent mixture of methanol:acetic acid (90 : 10 v/v) was passed until atenolol was no longer detected. The function of acetic acid was to break the bonds between the monomer and template [37]. The cleared imprinted monolithic column was characterised by the lack of absorption peaks from atenolol (maximum wavelength: 229 nm) detected by UV spectrophotometry.

### 3.3. Physical Characterisation

Physical characterisation was carried out to determine the physical shape of the polymer. Tests included permeability testing, FTIR, SEM, and SAA.

#### 3.3.1. Permeability Testing

Column permeability testing determines the ability of the mobile phase to flow through a column. Permeability testing was carried out by passing ethanol (viscosity 0.001095 Pa) through the monolithic column at a constant flow rate of 0.05 mL/minute, and back pressure data were recorded. The mobile phase flow produces a pressure that is inversely proportional to permeability based on the Darcy equation. The higher the pressure, the lower is the permeability, indicating that the flow of the mobile phase through the column is increasingly difficult.

There were differences in the column permeabilities, with permeability values of the MIP 1 and MIP 2 monoliths at a flow rate of 0.05 mL/minute being 24.01 mD and 23.03 mD, respectively. Moreover, in the NIP 1 and NIP 2 monoliths, the permeability values were 56.43 mD and 14.47 mD, respectively. Decreasing permeability values occur with an increase in the composition of the monomer because of the porogens found in a small polymer. The amount of porogen has an effect on the formation of pores; the less the porogen, the less pores are formed; thus, the density of the monolith increases [38]. Based on the permeability quality criteria described by Febrian et al., the four monolithic columns showed good permeability values; thus, their analytical performance was evaluated [39].

#### 3.3.2. SEM

**Figure 4:**
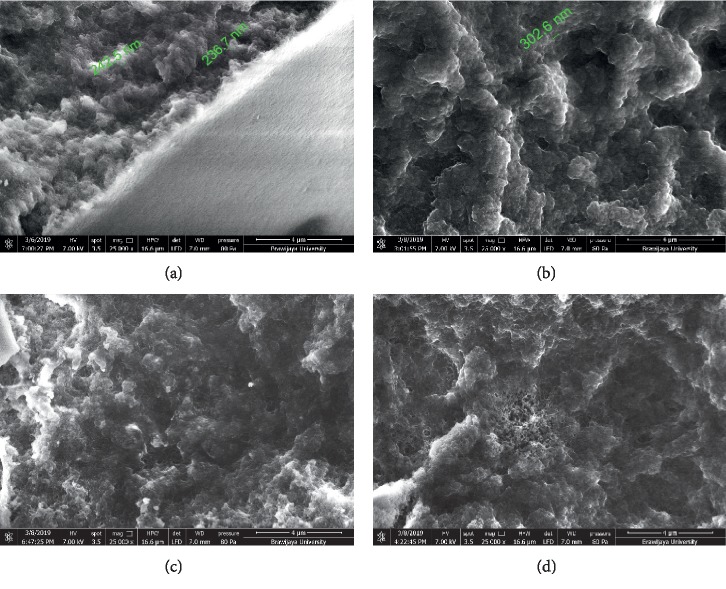
Polymer morphology as seen by SEM of the monolithic column MIP 1 (a); MIP 2 (b); NIP 1 (c); NIP 2 (d).

Characterisation by SEM serves to determine the surface morphology of the monolithic polymer. SEM coupled to EDS determines the components contained within the polymer. Based on Figure 4, the size of the MIP 1 globule was smaller (236.7 nm) than that of MIP 2 (302.6 nm) because of an increase in the number of crosslinkers [28]. The smaller macropores that are formed cause the density of the polymer to increase. The denser the polymer, the smaller is the pore, and the surface area and pore volume will also decrease, as seen in the SAA results shown in Table 2. The size of the micropores and mesopores in the globule cannot be seen; thus, characterisation testing is recommended using inverse size-exclusion chromatography (ISEC).

#### 3.3.3. FTIR

**Figure 5:**
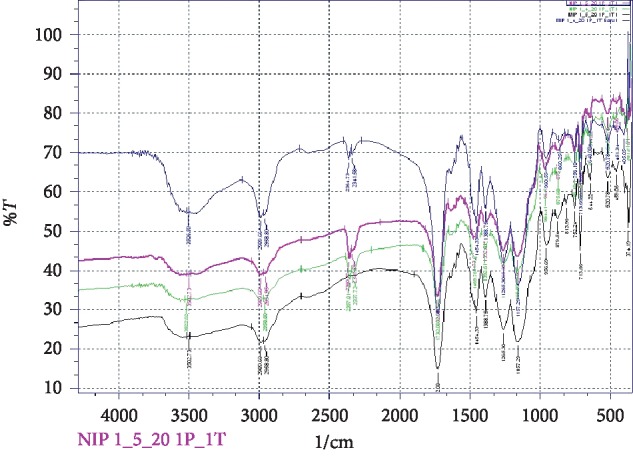
FTIR spectra of the imprinted monolithic columns (blue: MIP 1; black: MIP 2; green: NIP 1; violet: NIP 2).

Characterisation using FTIR spectroscopy determines the functional groups and types of bonds. Based on the FTIR results in Figure 5, the four polymers produced the same functional groups, one of which was hydroxyl (-OH) stretching as a carboxylic marker (-COOH) of MAA monomer molecules at a wave number of 3400−3650 cm^−1^. In addition, C-H stretching and–CH_2_ as markers of methylene groups from the MAA and EGDMA monomers were seen at wave numbers of 2900 cm^−1^ and 1400 cm^−1^. The strong intensity at a wave number of 1732 cm^−1^ shows carbonyl groups (C=O) from the MAA, EGDMA, and BPO monomers. The absorption peak at a wave number of 1673 cm^−1^ was a carbon double bond (C=C) from a vinyl group (−CH=CH_2_), but this overlapped with C=O absorption. This absorption peak shows that most MAA binds to EGDMA, and only a few remain unbound [40]. A vinyl group can be characterised by a double peak at the 100 cm^−1^ and 900 cm^−1^ wave numbers [41]. Based on the spectra, the four polymers did not show the presence of vinyl groups; therefore, it can be concluded that the polymerisation reaction of the four polymers was efficient.

#### 3.3.4. SAA

Characterisation of the imprinted monolithic column was carried out using SAA and the BET method to determine the pore diameter, surface area, and pore volume of the polymers. The surface area was determined by filling the polymer pores with inert N_2_ gas, which is adsorbed by the pore and forms the next monolayer of the multilayer. The destruction process ran at an output temperature of 130°C. Water molecules and the remaining impurities in the monolith will disappear at this temperature. A higher outgassing temperature (equal to or higher than the boiling point of water) will give the best results because the water molecules and the remaining impurities in the monolith evaporate entirely [20].

The exact molar ratio between the functional monomers of the template is very important in the formation of highly selective imprinted cavities. Increasing the average size of the pores and surface area increases the capacity of the template, thereby increasing rebinding, selectivity, and affinity [42]. Based on Table 2, MIP shows a larger surface area than NIP. The largest surface area was found in MIP 2, at 291.706 m^2^/g. The molar ratio between functional monomers and crosslinkers affects the polymer morphology. A small surface area and total pore volume were observed in relation to the higher molar ratio between crosslinkers and functional monomers (EGDMA : MAA = 5.0) in MIP 1 and NIP 1. Higher molar ratios cause reduced pore formation [43] and increased crosslinking density, increasing the density of the polymers [44].

A high proportion of macropores in monoliths is expected to provide convective mass transfer. Mesopores are also needed for interactions between analytes and stationary phases to provide a large surface area. In addition, the binding capacity of the target molecule is increased. The presence of micropores is not preferred because it can trigger peak widening, which further reduces separation efficiency. The ideal monolith has a balanced proportion of macropores and mesopores, resulting in a good binding capacity and efficient separation [28]. In the present study, the proportion of macropores to mesopores could not be determined precisely; thus, ISEC analysis must be used.

### 3.4. Characterisation of Analytical Performance

Performance characterisation was carried out on the four monolithic columns using the most optimal mobile phase, followed by determination of the chromatographic parameters. Analysis of atenolol in a previous study used isocratic elution with a mobile phase of methanol: 0.05% TEA pH 3 (15 : 85 v/v) [9]. Chromatographic profiles of the imprinted monolithic column using isocratic elution can be seen in Table 3.

Based on Table 3, the retention times of the three compounds were different, but the peaks generated were wide, as seen from the tailing factor values, which are greater than 2.0; thus, the three compounds were not well separated. In addition, the three analytes were quickly released because of the polar mobile phase. A mobile phase containing polar substances in the presence of acids or bases can weaken the target molecular bonds such that it escapes from the printed cavity in the stationary phase [45]. Moreover, an organic amine can reduce tailings at the peak, and methanol has been proven to be superior as a mobile phase modifier for the separation of several *β*-blockers [46]. Isocratic elution was not used for further testing because the retention times were too fast and the three compounds were not well separated.

Based on these results, optimisation was performed using gradient elution to better separate the three compounds. The main purpose of gradient elution is to modify retention times when the separation between compounds is inefficient [47]. Gradient elution of the mobile phase solvents (reservoirs A and B) was used. Reservoir A contained 100% acetonitrile, whereas reservoir B contained 90% methanol: 0.05% TEA (15 : 85 v/v) in acetonitrile.

Acetonitrile was chosen in reservoirs A and B because atenolol was practically insoluble; thus, the retention time would be longer because atenolol first binds to the template in the monolithic column. In addition, metoprolol and propranolol have different solubilities than atenolol, both of which are easily soluble in acetonitrile [19]. This is based on results using 100% acetonitrile mobile phase, with which there are differences in retention times and tailing factors between the three compounds, as can be seen in Table 4.

**Figure 6:**
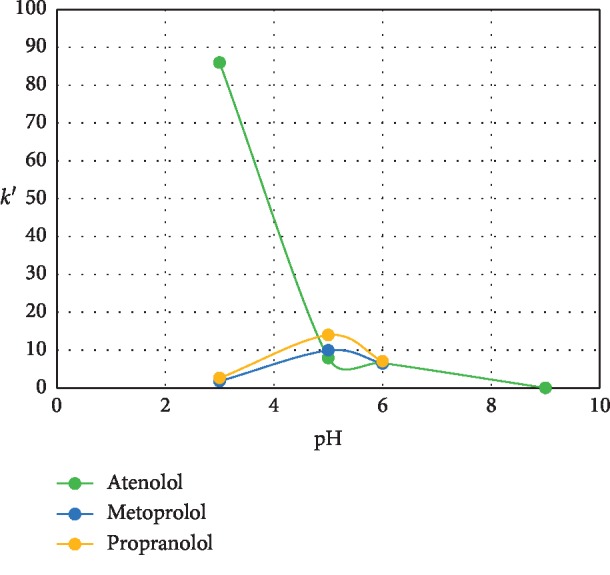
Effect of pH on the mobile phase towards the capacity factor. (Stationary phase: monolithic MIP 1 column; mobile phase: mixture of acetonitrile: (methanol: 0.05% TEA pH 3 (15 : 85)) 1 : 9 v/v).

The type and pH of the mobile phase affect the capacity factor value. The pH of the solvent can affect the solubility of ionised compounds. The solubility of weakly acidic compounds increases with increasing pH, whereas the solubility of weakly basic compounds increases with decreasing pH. Based on the pKa values, atenolol (pKa = 9.6), metoprolol (pKa = 14.09), and propranolol (pKa = 9.5) are weakly basic. The capacity factor values of the three compounds were almost the same when using the mobile phase of 100% acetonitrile (pH 4): atenolol (36.82), metoprolol (35.65), and propranolol (34.96). Therefore, the mobile phase was modified to a mixture of acetonitrile:(methanol:0.05% TEA (15 : 85)) (1 : 9 v/v), with variations in pH. These results can be seen in Figure 6.

At pH 9, atenolol was not eluted due to the degree of ionisation of both atenolol and MAA. The charge on MAA (pKa = 4.6) is very high since it is in the ionised form, while atenolol (pKa = 9.5) is partly in the ionised and non-ionised forms; therefore, a portion of atenolol remained on the column. At pH 5 and 6, the three *β*-blockers are in the ionised form, while MAA is in the ionised form because its pKa value is below the pH of the solution. There are differences in shape between MAA and the *β*-blocker analytes, causing bonding between MAA and the analytes to be weak and elute with the mobile phase [22]. At pH 3, the three compounds are in the ionised form because pH < pKa, but the capacity factor values of metoprolol and propranolol were lower than those of atenolol. At pH 3, the capacity factor of atenolol increased dramatically to almost 100 and was much higher than that of metoprolol and propranolol because of the imprinted effect. The atenolol anion is retained by MIP because of an electrostatic interaction with the carboxylic acid group in the polymer. The efficiency and peak symmetry of certain *β*-blockers increase with an increase in the mobility of the mobile phase, and the best results can be seen at pH 3 (phosphate buffer) [48].

The flow rate determines the retention time of an analyte, where a high flow rate causes the analyte to elute faster. However, too high a flow rate will cause the resulting pressure to be high, and the polymer inside the column may come out. Therefore, in the present study, the flow rate optimisation was carried out at 0.01 to 0.05 mL/minute.

Based on Table 5, the flow rate affected the retention time and the width of the resulting peak. The lower the flow rate, the longer atenolol took to elute, but a very wide peak could be seen from the tailing factor. The higher the flow rate, the faster atenolol eluted, but the resulting peak was narrow. A flow rate of 0.05 mL/minute produced a high pressure approaching 20 MPa; thus, the experiment was not carried out at this flow rate. The best flow rate of 0.04 mL/minute can be seen from a narrow peak; therefore, this was the flow rate used for subsequent analysis. According to this optimisation, the best mobile phase and HPLC conditions are displayed in Table 6.

**Figure 7:**
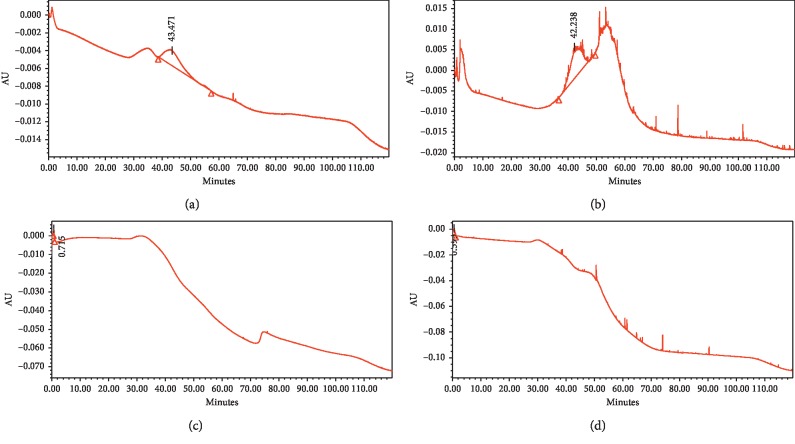
Chromatograms of atenolol standard solution using the monolithic imprinted column MIP 1 (a); MIP 2 (b); NIP 1 (c); NIP 2 (d).

Based on the chromatogram profiles in Figure 7, the peaks generated by tailing and width are caused by several factors such as the presence of nonspecific interactions. The template molecule is strongly maintained by MIP because there is a weak nonspecific recognition and interaction, resulting in a wider peak shape. In addition, the high crosslinking at the binding sites in MIP can inhibit the diffusion of analytes in the particles, resulting in peak widening. The concentration of standard solutions also affects the shape of the peak. At high concentrations, the nonspecific interactions between the analyte and the column increase; thus, the retention time is longer, and the peak tails more [45].

**Figure 8:**
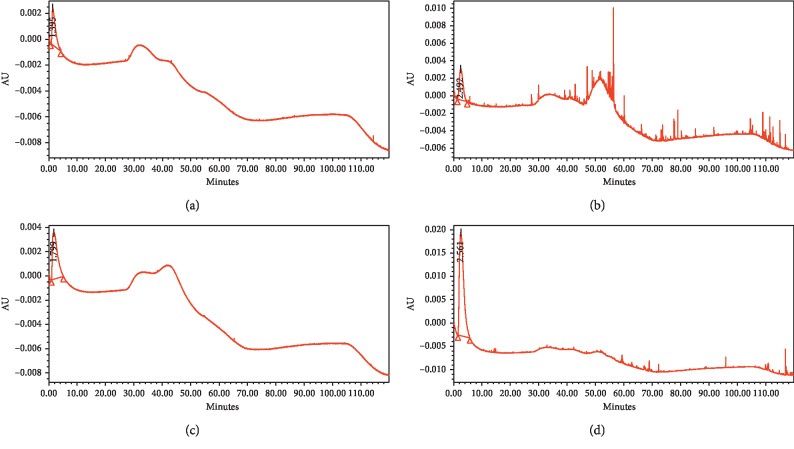
Chromatograms of metoprolol standard solution using the monolithic column MIP 1 (a); MIP 2 (b); and propranolol standard solution using monolithic column MIP 1 (c); MIP 2 (d).

In Table 7 and Figure 8, differences can be seen in the retention time generated using the MIP and NIP monolithic columns. MIP produced a longer retention time (43–45 minutes), whereas the NIP retention time was much faster (0.5–0.7 minutes). This is because the NIP does not possess a binding site in the monolithic column because there is no template and, therefore, no nonspecific bond between the analyte and the stationary phase [49]. The tailing factors of the MIP 2 imprinted monolithic column were smaller than those for MIP 1, but both were still within the tailing factor requirements of 2.0 [26]. The number of theoretical plates shows the efficiency of separation from a stationary phase; when the number of theoretical plates is higher, the better is the separation of the peak. Based on the terms of USP, the number of theoretical plates from a column must be > 2000 [26]. The imprinted monolithic column that fulfills these requirements is MIP 2 for the analysis of atenolol.

The longer retention time of standard atenolol when injected into the MIP 1 and MIP 2 columns is due to the presence of a specific molded cavity for atenolol that retains the analyte in the column. In contrast to the metoprolol and propranolol standards when injected into all four columns, atenolol had a faster retention time. Atenolol was practically insoluble in acetonitrile, whereas metoprolol and propranolol were soluble, which resulted in different retention times; metoprolol and propranolol eluted very quickly, whereas atenolol eluted slower.

The retention factor shows the level of attachment between analytes and stationary phases, ranging from 2 to 10. If the value of *k* < 2, there is no interaction between the analyte and the stationary phase, causing the analyte to elute faster. However, if the value of *k* >> 10, there is a strong interaction between the analyte and the stationary phase, and the analyte will elute slower [50]. The highest retention factor values were seen in MIP 1 and MIP 2 with atenolol analytes, at 85.54 and 89.05, respectively. The retention factor value was high because of the effects of imprinted MIP molecules, which have a specific recognition site for atenolol.

As shown in Tables 7 and 8, with respect to the peaks with a low retention time, there was a decrease in the area according to the concentration of the analyte. The peaks near the volume voids are often in bad shape or overlap with noise; thus, the area becomes smaller or larger, causing accuracy problems related to the concentration of the peak. Changes in the composition of the mobile phase will cause a decrease in resolution at the initial portion of the chromatogram, and as a result, the adjacent peaks will group. In addition, the balance time of the mobile phase will be slow. At the end of each gradient, the column must be rebalanced by passing the initial mobile phase [51].

### 3.5. Selectivity of the Imprinted Monolithic Column

The monolithic column was selectively tested using two *β*-blockers, namely, metoprolol and propranolol. Both drugs were chosen because they have functional groups similar to atenolol that can bind to MIP. The selectivity of the imprinted monolithic column was determined from the imprinting factor (IF) value obtained from the comparison of the retention factor (*k*) between MIP and NIP, as can be seen in Table 9.

The atenolol retention factor (*k*) of the MIP monolithic column was higher than that of the NIP monolithic column because the former has a specific binding site for atenolol. The highest IF values of the two monolithic columns were for atenolol (204.62 and 439.32), followed by metoprolol (3.36 and 12.84) and propranolol (1.27 and 8.58).

**Figure 9:**
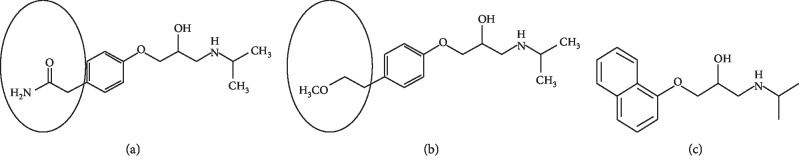
Chemical structure of *β*-blockers: atenolol (a); metoprolol (b); and propranolol (c) [52].

With respect to the chemical structure (Figure 9), metoprolol has a structural formula similar to that of atenolol, which only differs in the side groups. Atenolol has an amide side group, whereas metoprolol has a methoxy side group; therefore, the IF value of metoprolol is greater than that of propranolol.

**Figure 10:**
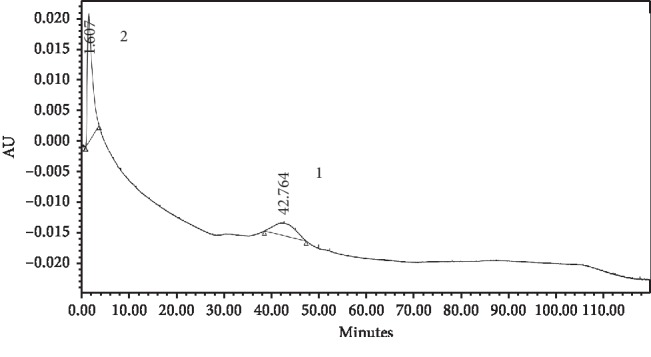
Chromatograms of atenolol (1) and its analogue (2) using the monolithic MIP 1 column.

Based on the selectivity factor (*α*) values shown in Table 9, the MIP 1 monolithic column had a greater *α* value than the MIP 2 monolithic column. The selectivity factor is also called the separation factor, with a value of *α* > 1 [26]. Selectivity can be seen from the resolution value of the three compounds when injected together at the same concentration. Resolution values indicate the degree of separation from each compound, with a value requirement greater than 1.5 [26]. The selectivity of the imprinted monolithic column can be seen in Table 9, and the resulting chromatogram can be seen in Figure 10.

Based on Table 10, the retention time of atenolol remained the same as in the previous test using a single atenolol solution, but the metoprolol and propranolol compounds did not separate at a retention time of 1.61 minutes. This can be seen from the AUC value of metoprolol and propranolol (1,429,517) almost doubling the AUC of atenolol (671,583). Previous testing of single solutions of metoprolol and propranolol showed that both analytes had adjacent retention times. The resolution value between atenolol peaks and the analogue compounds was 7.23, which meets the requirements for a resolution greater than 1.5.

### 3.6. Reproducibility Testing

Reproducibility testing aims to determine the inter- and intracolumn repeatability by calculating the percentage of relative standard deviations (% RSD) from retention time, area (AUC), capacity factor, and the number of theoretical plates of atenolol compounds. The RSD value for each of these parameters must be less than 2.0% [26]. The intercolumn reproducibility can be seen in Table 11.

Based on the intercolumn reproducibility shown in Table 11, the MIP 1 column had an RSD value below 2.0% for retention time, AUC, and capacity factor, whereas the MIP 2 column only had an acceptable repeatability value for the AUC parameter. The best repeatability value for the capacity factor parameter can be seen with the MIP 1 column, with an RSD value of 0.27%. Based on these results, the MIP 1 column was the best and was, therefore, tested for the analysis of atenolol in the serum.

The MIP 1 column was subjected to intracolumn repeatability testing by measuring atenolol standards three times and calculating the repeatability value (% RSD) of each chromatographic parameter. The intracolumn repetition results can be seen in Table 12, in which the MIP 1 column shows good intracolumn repeatability, with an RSD of all parameters less than 2.0% (USP–NF, 2017).

### 3.7. Application of the Imprinted Monolithic Column

The MIP 1 monolithic column had the best performance; thus, it was used to analyse atenolol in serum samples. The analysis was performed via spiked placebo recovery by adding standard atenolol solution to the serum followed by acetonitrile as a protein precipitant. The mixture was centrifuged at 2,000 rpm for 15 minutes at a temperature of 10°C [27]. The supernatant was measured by HPLC using the MIP 1 monolithic column under the best HPLC conditions, and the percentage recovery of the analyte was calculated. Requirements for percentage recovery rates of biological samples are in the range of 80−120% [53].

**Figure 11:**
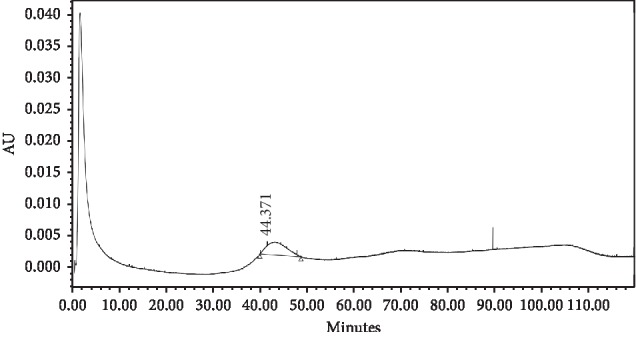
Chromatogram of atenolol in the serum using the MIP 1 monolithic column.

Based on Table 13, the percentage recovery rate of atenolol from the serum sample was 94.98% ± 4.43, which is within the required range of 80−120%; thus, the MIP 1 column can be used to analyse atenolol in the blood serum. The chromatogram from atenolol in the serum can be seen in Figure 11.

## 4. Conclusion

An imprinted monolithic column was constructed using two template:monomer:crosslinker ratios (1 : 4 : 20 and 1 : 5 : 20) with a mixture of 1 : 1 v/v propanol:toluene. Based on analytical performance characterisation, the best imprinted monolithic column was MIP 1 (1 : 4 : 20), with an RSD value < 2.0% in inter- and intracolumn reproducibility testing. The MIP 1 column can be used for the analysis of atenolol in blood serum samples, with an average percentage recovery of 94.88% ± 4.43.

## Figures and Tables

**Table 1:** Materials composition of the imprinted monolithic columns.

Polymer	Ratio (T : M : C)	Atenolol (T)	MAA (M)	EGDMA (C)	BPO (I)
MIP 1	1 : 4 : 20	0.45 mmol; 39.6 mg	1.8 mmol; 0.051 cm^3^	9 mmol; 0.567 cm^3^	1.55 mmol; 125 mg
MIP 2	0 : 4 : 20	—			
NIP 1	1 : 5 : 20	0.45 mmol; 39.6 mg	2.25 mmol; 0.064 cm^3^		
NIP 2	0 : 5 : 20	—			

**Table 2:** Pore structure characteristics of the monolithic columns.

Monolithic column	Surface area (m^2^/g)	Total pore volume (cm^3^/g)	Average pore diameter (nm)
MIP 1	200.216	0.577	11.541
MIP 2	291.706	1.36	18.651
NIP 1	159.579	0.572	14.393
NIP 2	249.785	1.53	24.421

**Table 3:** Chromatographic profile of the imprinted monolithic column with isocratic elution.

Standard	Retention time (*t*_R_) (minute)	Capacity factor (*k*′)	Tailing factor
Atenolol	0.85	0.69	2.24
Metoprolol	1.16	1.31	3.19
Propranolol	3.71	6.41	2.97

Stationary phase: monolithic MIP 1 column; mobile phase: methanol: 0.05% TEA pH 3 (15 : 85 v/v); flow rate: 0.04 mL/minute.

**Table 4:** Chromatographic profile of the imprinted monolithic column using the mobile phase acetonitrile.

Standard	Retention time (*t*_R_) (minute)	Capacity factor (*k*′)	Tailing factor
Atenolol	18.913	36.82	1.32
Metoprolol	18.326	35.65	1.37
Propranolol	17.981	34.96	1.61

**Table 5:** Effect of flow rate variation on the retention time of atenolol.

Flow rate (mL/minute)	Retention time (*t*_R_) (minute)	Tailing factor
0.01	4.45	4.11
0.02	2.24	3.49
0.04	0.75	1.34

Stationary phase: monolithic MIP 1 column; mobile phase: mixture of acetonitrile: (methanol: 0.05% TEA pH 3 (15 : 85)) 1 : 9 v/v.

**Table 6:** HPLC conditions for the imprinted monolithic column.

Parameter	Explanation
Mobile phase	Reservoir (A) : acetonitrile (ACN)
	Reservoir (B): 90% methanol:0.05% TEA (15 : 85) v/v in acetonitrile
Elution gradient	100−90% A for 28 minutes; 86.5–30.5% A for 30 minutes; 30−0% for 62 minutes
Flow rate	0.04 mL/minutes
Injection volume	3 *µ*L
Detector	UV 229 nm

**Table 7:** Chromatographic profile of atenolol standard solution using the imprinted monolithic column.

Monolithic column	*t*_R_ (minute)	AUC	*k*′	Tailing factor	Theoretical plates
MIP 1	43.371	660,428	85.94	1.72	262
MIP 2	45.026	1,028.435	89.05	0.58	2850
NIP 1	0.715	34,905	0.42	1.15	142
NIP 2	0.595	61,077	0.19	1.06	14.26

**Table 8:** Chromatographic profile of metoprolol and propranolol standard solutions.

Monolithic column	Standard	*t*_R_ (minute)	AUC	*k*′	Tailing factor	Theoretical plates
MIP 1	Metoprolol	1.395	235,68	1.78	3.17	5.97
	Propranolol	1.799	494,296	2.58	2.60	5.22

MIP 2	Metoprolol	2.665	282,829	4.33	1.34	22.9
	Propranolol	2.561	239,180	4.12	1.98	14.11

NIP 1	Metoprolol	0.764	24,119	0.53	1.06	7.6
	Propranolol	1.515	215,576	2.03	1.04	25.16

NIP 2	Metoprolol	0.344	17,241	0.31	30.78	18.66
	Propranolol	0.741	78,452	0.48	2.07	9.73

**Table 9:** Selectivity of the imprinted monolithic columns.

Standard	Monolithic column 1 (MIP 1)				Monolithic column 2 (MIP 2)			
	*k*_MIP_	*k*_NIP_	*IF*	*α*	*k*_MIP_	*k*_NIP_	*IF*	*α*
Atenolol	85.94	0.42	204.62		83.47	0.19	439.32	
Metoprolol	1.78	0.53	3.36	48.28	3.98	0.31	12.84	20.97
Propranolol	2.59	2.03	1.27	33.18	4.12	0.48	8.58	20.26

**Table 10:** Chromatographic profile of the *β*-blocker standard solution mixture.

Standard	*t*_R_ (minute)	AUC	*k*′	Resolution (Rs)
Metoprolol and propranolol	1.61	1,429.517	2.21	
Atenolol	42.76	671,583	84.53	7.23

**Table 11:** Intercolumn reproducibility of the imprinted monolithic column.

Parameter	Atenolol			
	*t*_R_	AUC	*t*_R_/AUC	*k*′
*MIP 1*				
Replication 1	43.37	660,428	6.5 × 10^−5^	85.94
Replication 2	43.64	666,152	6.6 × 10^−5^	86.28
Replication 3	43.41	670,433	6.4 × 10^−5^	85.83
Average	43.48	665,671	6.5 × 10^−5^	86.02
SD	0.14	5019,81	9.3 × 10^−7^	0.23
% RSD	0.33	0.75	1.42	0.27

*MIP 2*				
Replication 1	45.03	1,028.435	4.5 × 10^−5^	89.05
Replication 2	43.29	1,011.225	4.3 × 10^−5^	85.57
Replication 3	45.23	1,028.235	4.5 × 10^−5^	89.45
Average	44.51	1,022.632	4.4 × 10^−5^	88.02
SD	1.07	9878.97	1.1 × 10^−6^	2.13
% RSD	2.40	0.96	2.64	2.42

**Table 12:** Intracolumn reproducibility of MIP 1.

Standard	*t*_R_	AUC	*t*_R_/AUC	*k*′
Atenolol 1	43.53	658,206	6.6 × 10^−5^	86.05
Atenolol 2	43.36	642,419	6.7 × 10^−5^	85.70
Atenolol 3	43.69	650,011	6.7 × 10^−5^	86.37
Average	43.53	650,212	6.7 × 10^−5^	86.04
SD	0.16	7895.42	7.2 × 10^−7^	0.33
% RSD	0.38	1.21	1.08	0.40

**Table 13:** Analysis of atenolol in the serum using the monolithic column.

Sample	*t*_R_ (minutes)	AUC	*k*′	Recovery (%)
Serum 1	44.37	584,459	87.74	89.88
Serum 2	42.66	631,184	84.32	97.07
Serum 3	42.89	637,120	84.79	97.97
Average	43.31	617,587	85.62	94.98
SD	0.93	28,843.37	1.85	4.43

## Data Availability

The data used to support the findings of this study are included within the article.
